# Multiple sclerosis and risk of young-adult-onset Hodgkin lymphoma

**DOI:** 10.1212/NXI.0000000000000227

**Published:** 2016-04-14

**Authors:** Scott Montgomery, Mohammadhossein Hajiebrahimi, Sarah Burkill, Jan Hillert, Tomas Olsson, Shahram Bahmanyar

**Affiliations:** From Clinical Epidemiology and Biostatistics (S.M.), School of Medical Sciences, Örebro University, Sweden; Department of Epidemiology and Public Health (S.M.), University College London, UK; Clinical Epidemiology Unit (S.M., M.H.H., S. Burkill, S. Bahmanyar) and Centre for Pharmacoepidemiology (S. Bahmanyar), Department of Medicine, Solna, Karolinska Institutet; Department of Neurology (J.H., T.O.), Karolinska University Hospital Huddinge, Stockholm; Neuroimmunology Unit (J.H.), Department of Clinical Neuroscience and Centre for Molecular Medicine, Karolinska Institutet and Karolinska University Hospital, Solna, Sweden; and Faculty of Medicine (S. Bahmanyar), Golestan University of Medical Sciences, Gorgan, Iran.

## Abstract

**Objective:**

To determine whether there is an association between multiple sclerosis (MS) and young-adult-onset Hodgkin lymphoma (YAHL) as this will signal etiologic similarities relevant both to inherited characteristics and environmental exposures in childhood.

**Methods:**

Swedish general population registers identified a cohort of 29,617 with an MS diagnosis between 1968 and 2012, matched with a cohort of 296,164 without MS. Cox regression was used to assess the association of MS with subsequent YAHL (defined as onset between ages 15 and 39 years; n = 20), with adjustment, for age/period, sex, county of residence, and level of education.

**Results:**

The adjusted hazard ratio (and 95% confidence interval) for the association of MS with YAHL is 3.30 (1.01–10.73), resulting from 4 and 16 events in the MS and non-MS cohorts, respectively. All 4 of the YAHL diagnoses in MS occurred in women, and the association of MS with YAHL has a hazard ratio of 4.04 (1.17–13.94) among women. There was no notable association of MS with older-onset Hodgkin lymphoma.

**Conclusion:**

There may be common risks for YAHL and MS, consistent with an etiologic role in MS for early-life exposures, such as to infectious agents.

As multiple sclerosis (MS) and Hodgkin lymphoma (HL) share some epidemiologic characteristics, it has been suggested that there are common etiologic aspects.^1^ These characteristics include the putative role of patterns of exposure to microorganisms in childhood, such as Epstein-Barr virus (EBV).^1–5^ A previous study in Sweden of patients with MS (that included the majority of patients with MS involved in the current study) found a general reduction in cancer risk in MS, signaled by a hazard ratio (95% confidence interval) of 0.91 (0.87–0.95). There are some notable exceptions to the reduction in cancer risk, such as for brain tumors and urinary tract cancer.^6^ Despite the overall reduction of cancer risk in MS found in Sweden,^6^ a positive association may exist with HL, as indicated by an earlier Danish study that found evidence of familial clustering of MS and HL, with a non-statistically significant raised risk of HL in patients with MS.^1^ An association between MS and HL at the individual level would provide more evidence of a shared etiology, such that identification of specific exposures and mechanisms in one disease would merit investigation in the other.

HL has 3 age-defined phenotypes, but it is only young-adult-onset HL (YAHL)—ages 15–39 years—that is associated consistently with childhood markers of infectious exposure pattern^4,5^ and linked with MS in an earlier study.^1^ We therefore divided HL into its 3 phenotypes and assessed its occurrence among patients with MS using Swedish register data. Due to associations with environmental exposures in childhood in both MS and YAHL, it is our a priori hypothesis that MS is associated with YAHL but not the other HL phenotypes.

## METHODS

The Patient and MS registers provided information on patients diagnosed with MS in Sweden between 1968 and 2012. Details of hospital discharge diagnoses have been recorded in the Patient Register since 1964 and full national coverage was achieved in 1987.^7^ Information on patients with MS has been recorded in the MS Register since 1996.^8,9^ Prevalent patients with MS are on average younger in the MS Register than in the Patient Register as the former was founded more recently.^9^ Some 30,324 patients with MS were identified: 30,198 from the Patient Register and 10,807 from the MS Register (these sources are not mutually exclusive). Where patients were identified in both the MS Register and the Patient Register, their index date was taken as the earliest diagnosis of MS. The matching process was performed using information from the Total Population Register, which also provided dates of emigration and death for censoring. The design was that each patient with MS would be matched with 10 residents of Sweden without MS by sex, vital status at diagnosis, county of residence, and same year of birth. Matching with all 10 MS-free individuals was not achieved in 2 risk sets and 707 patients with MS were excluded as they could not be matched with any MS-free members of the general population. A total of 14 patients with MS and 117 without were excluded as they had a diagnosis of HL prior to study entry. Thus, for the analysis there were 29,603 patients with MS and 296,047 without.

HL (with YAHL defined as ages 15–39 years, as in our previous studies^4,5^) was identified through the Cancer Register, which began in 1958 and was considered to have largely complete coverage of malignant cancer diagnoses,^10^ as reporting is mandatory. All cancer diagnoses are back-coded to ICD-7, so here HL was identified using code 201. Census data specified the total number of years in full-time education to indicate level of educational attainment. Linkage between data sources used the identity number issued to all residents in Sweden.

### Statistical analysis.

The association of MS with HL was investigated using Cox regression. Entry into the study was at the first MS diagnosis and at an equivalent time for members of the non-MS cohort, follow-ended at diagnosis of HL, death, emigration, December 31, 2012, whichever occurred first. Separate models were used for the HL phenotypes, with follow-up ending at a maximum age of 39 years for YAHL. Childhood-onset HL was not examined, as few of those in the study were young enough. The models were adjusted for matching characteristics and number of years in full-time education, all modeled as series of binary dummy variables. Further stratification by sex was performed.

### Ethics approval.

The Karolinska Institutet ethics committee approved this study.

## RESULTS

The sex distribution is consistent with the female excess in MS and the age distribution reflects the tendency to early adult onset in MS (table 1). As these measures and year of study entry are matching factors, there are no notable differences in distribution between the cohorts. There are no substantial differences for number of years in full-time education. A higher proportion of those with MS have a diagnosis of YAHL and this is more fully described in table 2. MS is associated with a raised risk of all HL, but this is only statistically significant among women (table 2). After adjustment, a statistically significant raised risk for HL among patients with MS was observed for YAHL, but not older-onset HL. The association between MS and YAHL is entirely explained by diagnoses among women.

**Table 1:**
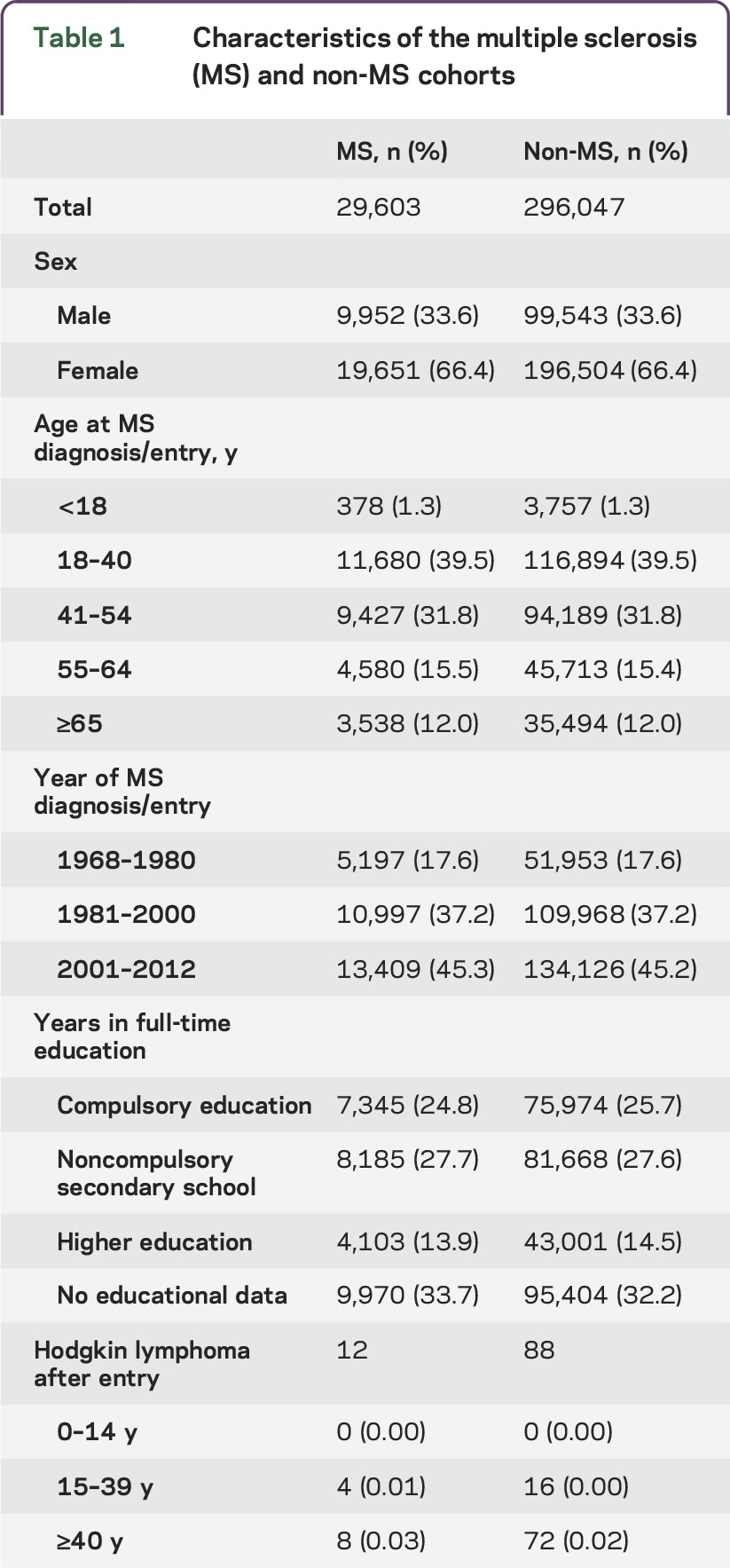
Characteristics of the multiple sclerosis (MS) and non-MS cohorts

**Table 2:**
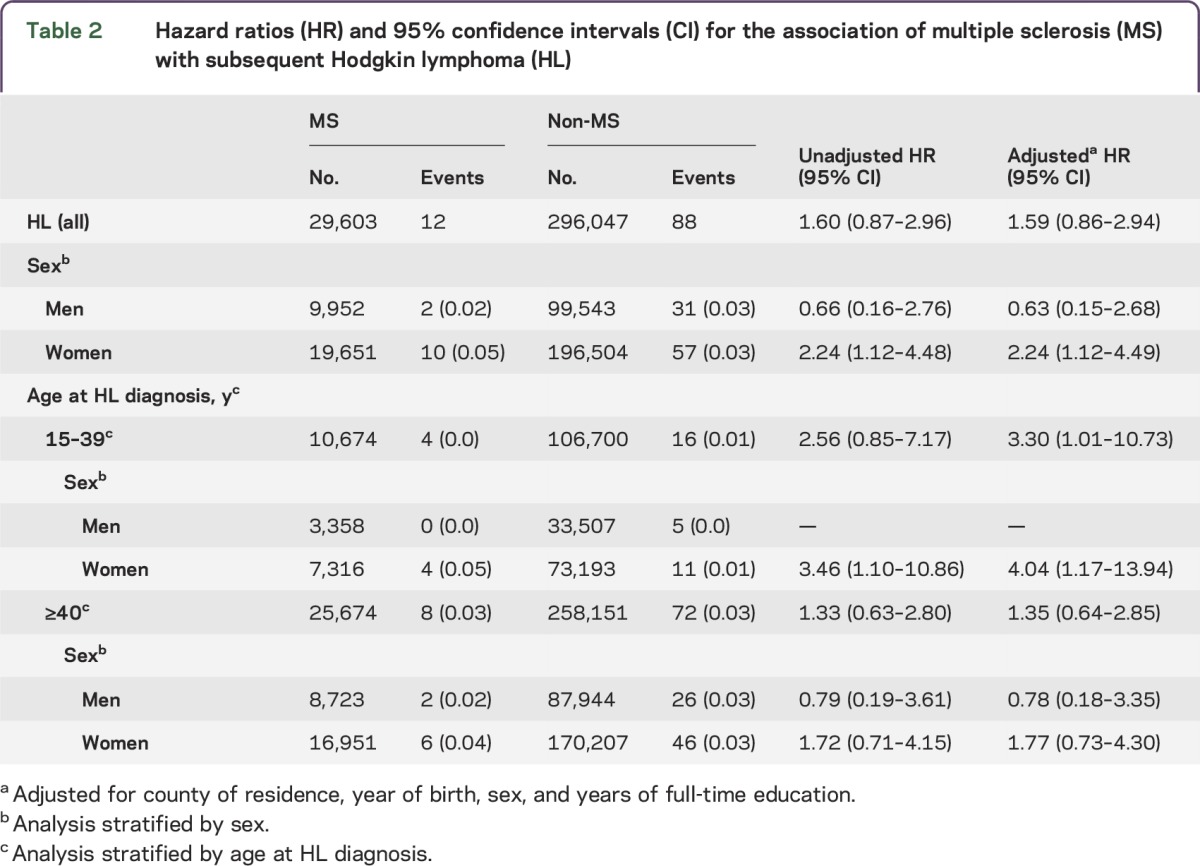
Hazard ratios (HR) and 95% confidence intervals (CI) for the association of multiple sclerosis (MS) with subsequent Hodgkin lymphoma (HL)

## DISCUSSION

The positive and statistically significant association of MS with subsequent YAHL is consistent with shared environmental or inherited risks for both diseases, potentially indicating etiologic processes relevant to MS. Due to associations with environmental exposures in childhood in both MS and YAHL, it was our a priori hypothesis that MS is associated with YAHL but not the other HL phenotypes, consistent with a Danish study that identified a familial association of MS with YAHL,^1^ even though the association in the earlier study was not statistically significant for individuals with both diseases possibly due to lower statistical power. Our study suggests the association exists most notably in female patients: this may be a chance finding as the majority of those with MS are women (there is lower statistical power to detect an association among men), or because of sex-specific differences in susceptibility to a risk for both diseases.

It has been proposed that shared susceptibility to MS and HL may in part be due to genetic factors such as the HLA DR2 allele,^1^ but it may also be because MS and YAHL (although not the other HL phenotypes) are both associated with markers of pattern of childhood exposure to microorganisms,^2–5^ suggesting an infectious etiology that is, at least in part, shared. EBV is implicated as a risk for both diseases,^1^ but as the markers of pattern of infection used previously are nonspecific, such as presence of siblings or household crowding,^2–5^ other infectious agents could be involved. As adjustment for educational level—which is associated with childhood circumstances—has little effect, significant confounding by socioeconomic factors is unlikely.

This study has several potential limitations, in addition to its inability to identify specific childhood exposures that underlie the association, and we do not have information on some potentially relevant personal characteristics such as ethnic origin. The data structure was designed to look at outcomes following an MS diagnosis, limiting our ability to look at childhood-onset HL due to the age profile of MS onset. We cannot rule out the possibility that MS disease activity or treatment increases the risk of YAHL, but as a study identified familial clustering of the 2 diseases,^1^ this is unlikely. During the earlier portion of the study period, MS was only identified through inpatient diagnoses, which is likely to result in bias towards inclusion of those with more severe disease. We did not exclude members of the non-MS cohort who had demyelinating diseases other than MS. While the absolute number of individuals with these diseases may be relatively low, their inclusion in the comparison cohort could have biased our estimates towards the null if these demyelinating diseases share risk factors with MS that are also relevant to YAHL etiology. The differences between HL phenotypes may only be captured crudely as defined by onset age and the definition can vary between studies, so we have used the same definition of YAHL as in our previous studies.^4,5^ Despite the size of our study, the number with both MS and YAHL is small, reflecting the relatively rare occurrence of HL and that patterns of exposure that constitute risks for these diseases are not identical, even though common factors are involved.

The association of MS with YAHL indicates shared inherited or acquired risks for these diseases, which are not necessarily mutually exclusive. Although the study cannot demonstrate which exposures are responsible, they might include some previously identified shared putative factors, such as pattern of childhood infectious exposures.

## GLOSSARY

EBV: Epstein-Barr virus
HL: Hodgkin lymphoma
ICD: *International Classification of Diseases*
MS: multiple sclerosis
YAHL: young-adult-onset HL
